# Mosquitoes in an Urban Zoo: Identification of Blood Meals, Flight Distances of Engorged Females, and Avian Malaria Infections

**DOI:** 10.3389/fvets.2020.00460

**Published:** 2020-08-21

**Authors:** Josué Martínez-de la Puente, Ramón Soriguer, Juan Carlos Senar, Jordi Figuerola, Rubén Bueno-Mari, Tomás Montalvo

**Affiliations:** ^1^Estación Biológica de Doñana (EBD-CSIC), Sevilla, Spain; ^2^CIBER Epidemiología y Salud Pública (CIBERESP), Madrid, Spain; ^3^Evolutionary and Behavioural Ecology Research Unit, Museu de Ciències Naturals de Barcelona, Barcelona, Spain; ^4^Laboratorios Lokímica, Departamento de Investigación y Desarrollo (I+D), Valencia, Spain; ^5^Agencia de Salud Pública de Barcelona, Consorci Sanitari de Barcelona, Barcelona, Spain

**Keywords:** *Aedes albopictus*, avian *Plasmodium*, *Culex pipiens*, malaria, vectors

## Abstract

Zoological gardens are home to a large number of vertebrate species and as such are suitable sites for both mosquito breeding and maintenance. They are excellent places for entomological studies of mosquito phenology, diversity, and blood-feeding patterns, as well as for xenomonitoring. During 2016, we sampled mosquitoes in Barcelona Zoo and used molecular methods to determine their blood-feeding patterns and the prevalence and diversity of avian malaria parasites. We also estimated the flight distance of engorged mosquitoes in the area. Overall, 1,384 adult *Culex pipiens* s.l., *Culiseta longiareolata*, and *Aedes albopictus* were captured. Birds dominated the diet of *Cx. pipiens* s.l. (*n* = 87) and *Cs. longiareolata* (*n* = 6), while humans were the only blood-meal source of *Ae. albopictus* (*n* = 3). Mosquitoes had a mean flight distance of 95.67 m after feeding on blood (range 38.71–168.51 m). Blood parasites were detected in the abdomen of 13 engorged *Cx. pipiens* s.l., eight of which had fed on magpies. Four *Plasmodium* lineages and a single lineage of the malaria-like parasite *Haemoproteus* were identified. These results suggest that *Cx. pipiens* s.l. is involved in the local transmission of avian *Plasmodium*, which potentially affects the circulation of parasites between and within wildlife and enclosed animals. Vigilance regarding possible mosquito breeding sites in this zoo is thus recommended.

## Introduction

Mosquitoes transmit a diversity of vector-borne pathogens affecting humans, livestock, and wildlife (1, 2). In addition to native species, invasive mosquitoes such as alien *Aedes* mosquitoes are involved in the circulation of both imported and locally circulating pathogens (3). This is the case of the invasive Asian tiger mosquito *Aedes albopictus*, which is associated with the local transmission of pathogens such as filarioid worms (e.g., *Dirofilaria* spp.), protozoa (e.g., avian malaria parasites), and viruses (e.g., Dengue virus) (4–6). This species has a broad global distribution and is present in countries outside its native range in America, Europe, Oceania, and Africa (7). In Spain, *Ae. albopictus* was first recorded in 2004 in Catalonia and since then has progressively colonized different parts of this region (8).

Zoos and wildlife parks with non-autochthonous and stabled fauna are excellent sites for studying the ecology and epidemiology of vector-borne pathogens, as studies of sand flies (9, 10), biting midges *Culicoides* (11), and mosquitoes (12–15) have previously shown. Given that captive animals are housed in known locations, the flight distances of captured insect vectors containing blood from these animals can be accurately estimated (10). In addition, these areas are frequented by human visitors and there are often both freely moving animals and animals maintained in captivity, all of which are potentially exposed to the transmission of various pathogens (15). This is especially the case of avian malaria parasites of the genus *Plasmodium*, a group of haemosporidians that naturally circulates between birds and mosquitoes (16) that can severely affect the health and survival of birds in zoos and recovery centers (17–22). However, in spite of their veterinary importance, the transmission dynamics of mosquito-borne avian *Plasmodium* in these particular ecosystems are still poorly understood [e.g., (12, 15, 23, 24)].

Here, we sampled mosquitoes in Barcelona Zoo, a site with a great diversity of mosquito and bird species, many of which are infected by avian malaria parasites (25). We employed a comprehensive approach based on the analysis of mosquito blood-feeding patterns, xenomonitoring (defined as the identification of pathogens in mosquito vectors), and flight distances of engorged females. We used molecular methods to identify the blood-meal sources of both native and alien engorged mosquitoes and to screen for the presence of avian *Plasmodium* in the abdomens of engorged mosquitoes to assess contact rates between potential vectors and parasites in the area.

## Methods

### Study Area, Mosquito Sampling, and Species Identification

**Figure 1:**
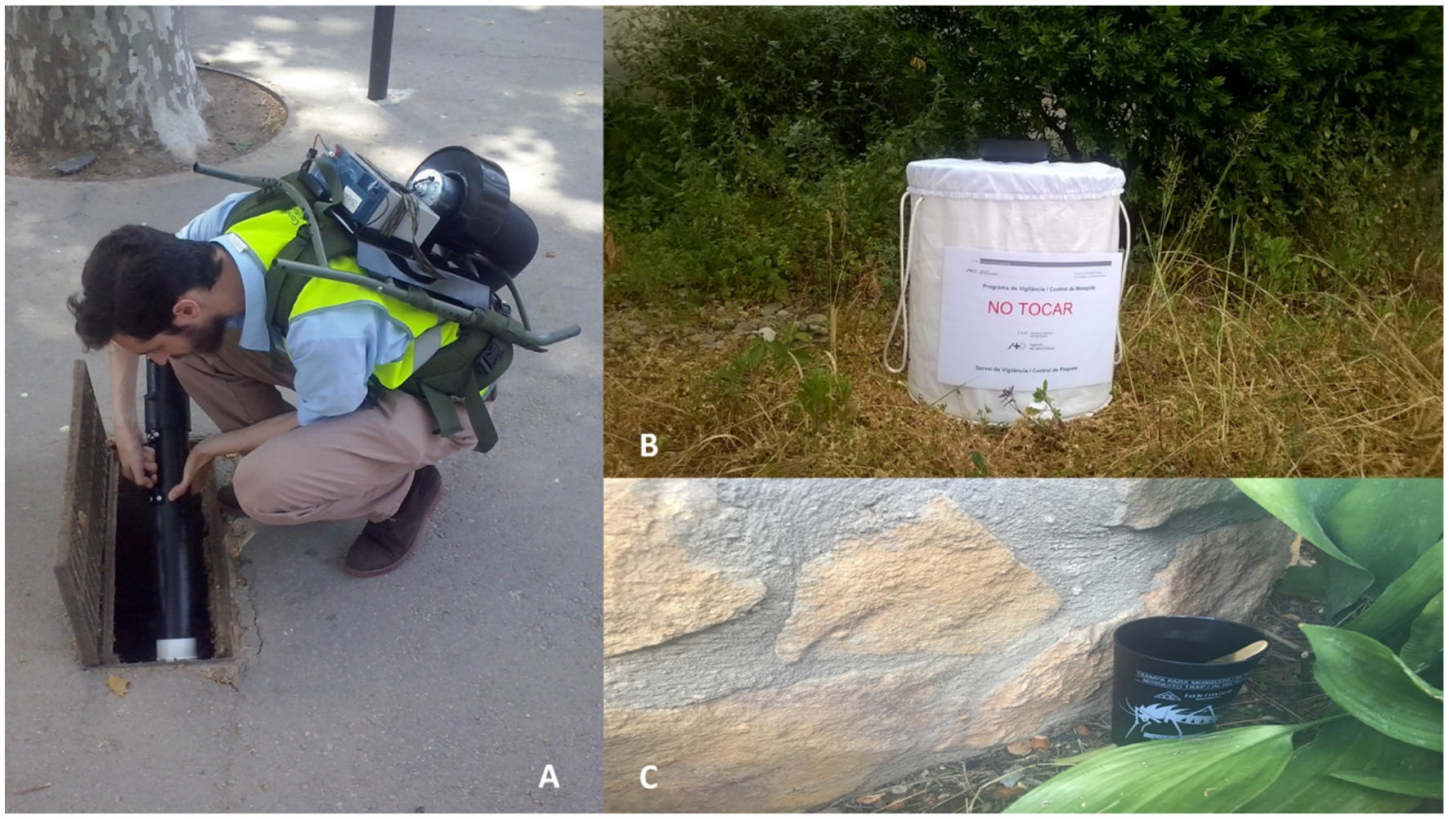
Sampling methods used in this study including direct aspiration in mosquito resting areas **(A)**, BG-Sentinel traps **(B)**, and ovitraps **(C)**.

Mosquitoes were collected in June–November 2016 in Barcelona Zoo using both passive and active trapping techniques (Figure 1). BG-Sentinel traps (Biogents GmbH, Regensburg, Germany) were installed for 24 h in three different sites in the zoo, namely, the aviary, the farm, and the terrarium. These sites were selected according to criteria related to host proximity, distance between traps, and cover and protection for the traps. Every 2 weeks, mosquitoes were sampled using BG-Sentinel traps baited with CO_2_ during 24 h. In addition, we used entomological aspirators (Improved Prokopack Aspirator, Mod. 1419, John W. Hock Company, FL, USA, and CDC Backpack Aspirator Mod. 2846, BioQuip, CA, USA) to collect mosquitoes resting on vegetation, bins, and animal cages. Aspirations were performed in six pre-established sites distributed throughout the whole zoo (13.5 ha) for 5 min in each area to standardize active adult mosquito sampling and increase the overall number of blood-fed mosquitoes collected. Additionally, 10 standard ovitraps (350 ml water capacity) were installed and monitored weekly to obtain information on mosquito phenology. Larval samplings were conducted occasionally to check for the presence/absence of other species that had not been collected by the above-described methodologies. Mosquitoes were identified using morphological keys (26); females containing any remains of blood meals in their abdomens were stored individually at −80°C until subsequent molecular analyses.

### Molecular Identification of Blood-Meal Sources and Parasites in Engorged Mosquitoes

The abdomen of each engorged mosquito was separated from the head–thorax using sterile tips on Petri dishes. Genomic DNA was isolated from these abdomens using Maxwell® 16 LEV System Research Kit (Promega, Madison, WI). The protocol described by Alcaide et al. (27) involving the amplification of the barcoding region of the cytochrome oxidase subunit 1 (COI) gene was used to identify the blood-meal origin of mosquitoes [see also (28) for the adaptation of the protocol for *Ae. albopictus*]. The presence of avian *Plasmodium*/*Haemoproteus* parasites was screened for following Hellgren et al. (29). Amplified fragments were sequenced using the Macrogen Inc. facilities (Amsterdam, The Netherlands), and the resulting sequences were edited using Sequencher™ v4.9 (Gene Codes Corp., 1991–2009, Ann Arbor, MI 48108, USA). Blood-meal sources were identified by comparing sequences obtained with those deposited in public-access databases (GenBank DNA, National Center for Biotechnology Information Blast and the Barcode of Life Data Systems), bearing in mind the species potentially present in the study area. Parasite lineages and, whenever possible, their respective morpho-species were identified by Blast comparison with sequences in GenBank and/or MalAvi (30).

### Analyses of Flight Distances of Blood-Fed Female Mosquitoes

**Figure 2:**
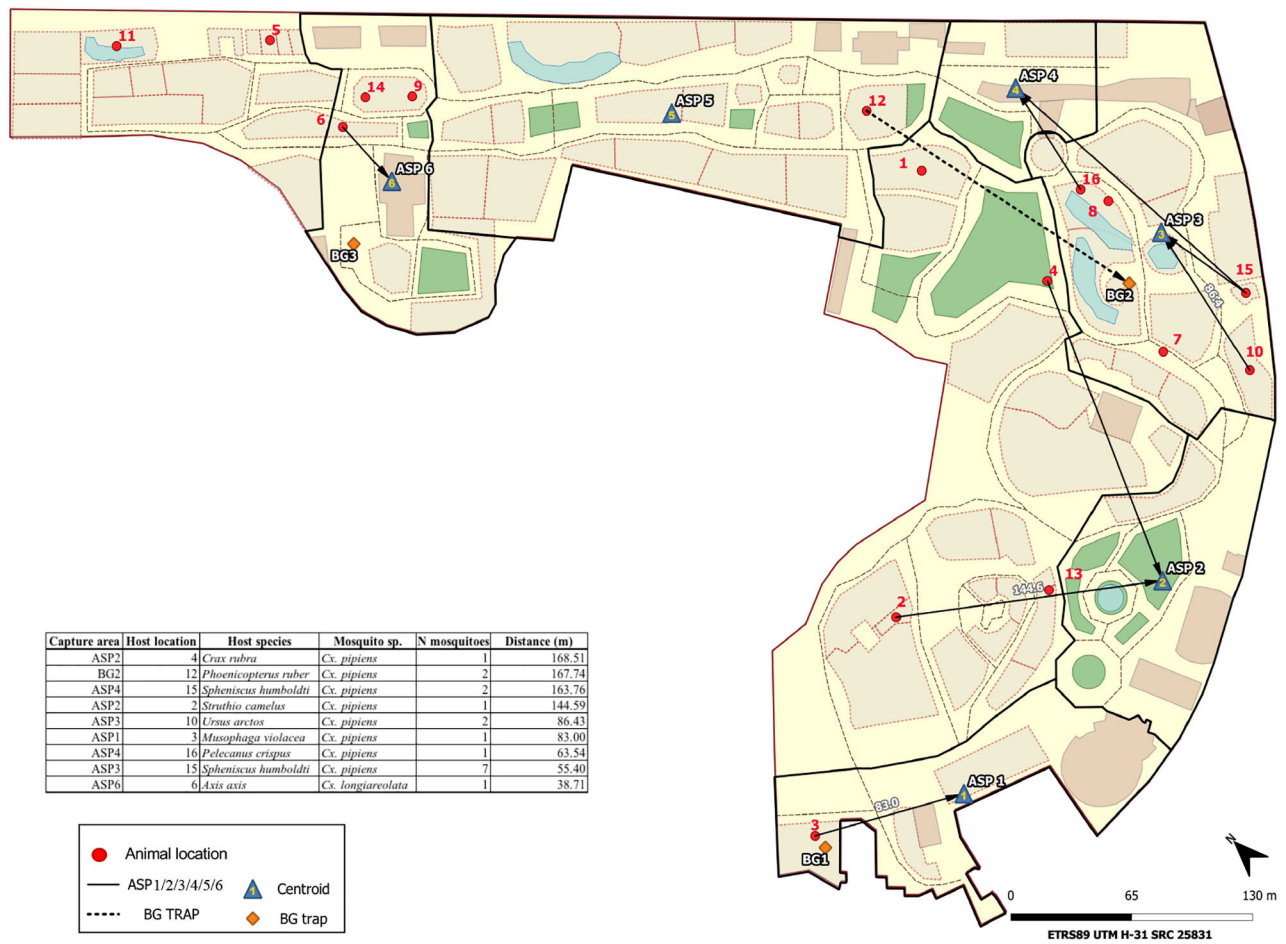
Study area and sampling sites of mosquitoes. The post-flight distances of engorged female mosquitoes are shown.

We estimated the minimum flight distances of mosquitoes using data concerning where mosquitoes were captured and the locations of caged host species (Figure 2). Mosquito trapping areas and enclosures within the zoo of identified vertebrate host species were georeferenced. Only vertebrate species in enclosures in the zoo were considered in the analyses. Freely moving hosts (e.g., humans) were excluded from the analyses. Based on host identification, distances between the centroids of the areas enclosing animals and mosquito sampling points (BG-Sentinel stations or entomological aspiration points) were measured to calculate flight distances. Analyses were carried out with QGIS 3.12 (QGIS Development Team. http://qgis.osgeo.org).

## Results

We trapped 1,384 adult *Culex pipiens* s.l. (*n* = 733; 52.96%), *Ae. albopictus* (*n* = 490; 35.40%), and *Culiseta longiareolata* (*n* = 161; 11.63%). In total, 63.29% (*n* = 876) of these mosquitoes were collected using entomological aspirators and the remaining 36.71% (*n* = 508) using BG-Sentinel traps. Catches of *Cx. pipiens* s.l. peaked in June–July, while those of *Ae. albopictus* adults peaked in September–October; these results were supported by both direct collection by entomological aspirators and the BG-Sentinel traps. In addition, we collected 9,658 *Ae. albopictus* eggs, with a maximum in July–October and a peak in August (3,965 eggs).

In all, 137 (9.90%) mosquitoes had some blood remains in their abdomens, of which the vertebrate hosts of 86 (62.32%) were successfully identified (birds and mammals) (Table 1). Birds dominated the diet of *Cx. pipiens* s.l. (92.21%; *n* = 77). The three identified *Ae. albopictus* blood meals came from humans, while the six blood meals from *Cs. longiareolata* corresponded to birds. Post-blood-feeding flight distances were calculated for the 18 mosquitoes that had fed on enclosed animals (17 *Cx. pipiens* s.l. and a single *Cs. longiareolata*) (Figure 2). For *Cx. pipiens* s.l., the mean post-blood feeding flight distance was 99.02 m, with a maximum of 168.51 m. The only distance measured for *Cs. longiareolata* was 38.71 meters. Overall, 13 (9.49%) out of the 137 engorged mosquitoes analyzed were positive for the presence of parasites (Table 1). All the parasite lineages found in this study coincided with *Plasmodium* lineages: Delurb4 (*n* = 1), Delurb5 (*n* = 3), Syat05 (*n* = 1, corresponding to *P. vaughani*), SGS1 (*n* = 1, corresponding to *P. relictum*), and the *Haemoproteus* lineage hCIRCUM04 (*n* = 5, also called BLUTI9). Sequences corresponding to each of the five lineages identified in this study were deposited in GenBank (MT568928–MT568932). In addition, one *Plasmodium* parasite and one *Haemoproteus* parasite were detected in mosquitoes, although the low quality of these sequences did not allow us to accurately identify their parasite lineages. Twelve out of the 13 positive mosquitoes corresponded to *Cx. pipiens* s.l. A single *Cs*. *longiareolata* was positive for *Haemoproteus* lineage hCIRCUM04.

**Table 1:** Vertebrate hosts of mosquitoes trapped in the zoological garden of Barcelona during 2016.

**Host**	***Ae. albopictus***	***Cs. longiareolata***	***Cx. pipiens***	**Parasites**
**Birds**				
*Ardea cinerea*			12	
*Ardea* sp.		1		
*Bubulcus ibis*		1	18	
*Columba livia*			1	*Haemoproteus* sp. (1)
*Corvus monedula*			1	
*Egretta garzetta/*sp.			4	
*Crax rubra*			1	
*Musophaga violacea*			1	
*Myiopsitta monachus*			3	
*Passer domesticus*		1		
*Pavo* sp.			2	
*Pelecanus* sp.			1	
*Phoenicopterus ruber*			2	*P. vaughani* SYAT05 (1)
*Pica pica*		2	8	*Haemoproteus* hCIRCUM04 (3), *Plasmodium* Delurb5 (3), *Plasmodium* Delurb4 (1), *Plasmodium* sp. (1)
*Spheniscus humboldti*			9	*P. relictum* SGS1 (1)
*Streptopelia decaocto*			7	*Haemoproteus* hCIRCUM04 (1)
*Struthio camelus*			1	
**Mammals**				
*Axis axis*		1		
*Canis lupus*			1	
*Homo sapiens*	3		3	
*Ursus arctos*			2	

*The parasite lineages identified in mosquitoes fed on each animal are shown. One mosquito with an unidentified blood meal was positive for Haemoproteus (hCIRCUM04)*.

## Discussion

Infections by *Plasmodium* parasites and related haemosporidians are commonly found in birds in zoos (31) and can lead to illness and/or lethal diseases (20–22). These cases usually occur in immunologically naïve species such as penguins originating from areas where the parasites circulating in zoos are not present. These animals are frequently bitten by competent vectors of avian malaria parasites, especially of the genus *Culex* (18), and largely suffer the costs of infections (17, 19). Interestingly, we found that a number of *Cx. pipiens* s.l. had fed on Humboldt penguins (*Spheniscus humboldti*), including mosquitoes with *P. relictum*. Different treatments and prophylactic protocols are applied to penguins to minimize the parasite-induced illness (18), as is also common practice in the study area.

Our results regarding the blood-feeding pattern of mosquitoes support the hypothesis that *Cx. pipiens* s.l. females feed mainly on birds (6, 28, 32). Despite the extremely low sample size, we also found support for the ornithophilic behavior of *Cs. longiareolata*; however, *Ae. albopictus* fed exclusively on humans. This latter species is a nuisance for visitors, especially in summer when it is commonest. These results provide valuable information regarding the blood-feeding sources of these species, which is especially relevant in the case of *Ae. albopictus* as this subject has only to date been investigated at a handful of studies in Europe (6, 25, 28, 33). Identification of the blood-meal sources of mosquitoes in zoos allows researchers to investigate additional aspects of vector ecology such as the flight distances of engorged females. Greenberg et al. (13) recorded an average distance of 106.7 m for mosquitoes of the genus *Aedes* (*Ae. vexans*), *Culex* (*Cx. Quinquefasciatus*, and *Cx. tarsalis*), and *Culiseta* (*Cs. inornata*) in USA. Similar values were reported by Tuten et al. (34) in zoos from USA, where *Anopheles* and *Culex* mosquitoes flew a mean distance of 94.1 m after feeding. Ejiri et al. (12) found that the post-blood-meal flight distance of *Cx. pipiens pallens* lay in the range 10–350 m in Japan. Heym et al. (14) estimated the mean post-blood-meal flight distance of *Cx. pipiens* (biotype *pipiens*) in two zoos in Germany as 132.2 and 362.3 m. In this study in a Mediterranean area, engorged *Cx. pipiens* s.l. and *Cs. longiareolata* were captured in average at 95.67 m from their hosts, with a maximum of 168.51 m for *Cx. pipiens* s.l. It is worth highlighting that only a single female of *Cs. longiareolata* was found to have blood from zoo animals and so further studies targeting this species are still required. We were not able to calculate the flight distances of *Ae. albopictus* due to that fact that in this study it fed exclusively on humans.

Our results suggest that encounters between infected birds and competent mosquito vectors frequently occur in the area, which probably facilitates the transmission of avian *Plasmodium* within this zoo (12, 15, 23, 24). Previous studies in the Iberian Peninsula have identified avian *Plasmodium* in mosquitoes of the genera *Culex* and *Aedes* (25, 32, 35, 36), and of them, *Cx. pipiens* probably plays a key role in the transmission of avian *Plasmodium* due to its wide distribution (1), competence for the transmission of different avian *Plasmodium* lineages/species (16, 37), and its ornithophilic behavior (6, 32). A previous study in the same area also identified the presence of avian *Plasmodium* in *Cx. pipiens* s.l. pools (25). Nevertheless, the molecular identification of parasite DNA in mosquitoes does not necessarily imply vector competence (38) and so we were not able to assess the competence of these mosquitoes for avian malaria transmission. This is the case, above all, of *Haemoproteus* parasites, a parasite genus transmitted by *Culicoides* spp. and louse flies (16), that cannot be effectively transmitted by mosquitoes.

## Conclusion

Barcelona Zoo is a suitable site for the development and maintenance of both native and alien species of mosquitoes. Based on their blood-feeding patterns, *Cx. pipiens* s.l. probably play a role in the local transmission and spread of mosquito-borne pathogens. This ornithophilic mosquito is a well-known vector of avian *Plasmodium* parasites. Thus, *Cx. pipiens* s.l. and the parasites transmitted may have an impact on the health of caged birds and so very likely represent a veterinary concern. In addition, magpies are common hosts of mosquitoes in the area and it is likely that they act as *Plasmodium* spp. reservoirs since high parasite prevalence has been found in the mosquitoes that feed on this bird species.

## Data Availability Statement

All data analysed during this study are included in this published article, any further information is available from the corresponding author on reasonable request.

## Ethics Statement

Ethical approval was not required for this study according to national/local legislation because mosquitoes are not protected by any law. Written informed consent was obtained from the individual for the publication of any potentially identifiable images or data included in this article.

## Author Contributions

JM, JF, RB-M, and TM designed the study. JS, RB-M, and TM performed the fieldwork. JM, RS, JF, RB-M, and TM analyzed the samples and data. RS, JF, RB-M, and TM contributed with reagents. JM drafted the first version of the manuscript. All authors read and approved this manuscript.

## Conflict of Interest

The authors declare that the research was conducted in the absence of any commercial or financial relationships that could be construed as a potential conflict of interest.
